# Remarks on the Constitution of the Medical Department of the British Army, &c.

**Published:** 1843-01-01

**Authors:** 


I. REMARKS ON THE CONSTITUTION OF THE MEDICAL DEPARTMENT OF THE BRITISH ARMY, WITH A DETAIL OF HOSPITAL MANAGEMENT, AND AN APPENDIX. By *Robert Jackson,* M.D. London, 1803.

II. A SYSTEM OF ARRANGEMENT AND DISCIPLINE. FOR THE MEDICAL DEPARTMENT OF ARMIES. By *Robert Jackson,* M.D. London, 1805.

III. FIFTH REPORT OF THE COMMISSIONERS OF MILITARY ENQUIRY—ARMY MEDICAL DEPARTMENT. Ordered to be Printed, 26th January, 1808.

IV. NOTES ON THE PRESENT CONDITION OF THE MEDICAL DEPARTMENT OF THE INDIAN ARMY. By *J. R. Martin,* Esq. Surgeon on the Bengal Establishment.

IN placing the titles of the above works as a heading to our present article, we do not by any means intend to give either a review or an analysis of their contents. What we propose is, to place before our readers a rapid sketch of the Administrative History of the British Army Medical Department, as well as of that of the Indian Army; noting the causes that brought about their respective improvements. We think such an exposé, however brief, will prove an useful record; for it is only by an acquaintance with preceding errors that we can effectually apply their preventives, and obviate their results.

Who is there that considers the vast debts due by the science of medicine and surgery to the medical officers of our fleets and armies, that will deny to this subject a paramount importance? Who is there that regards the fame and usefulness of men like Pringle, Blane, Robert Jackson, Macgrigor, Fergusson, Hennen, Guthrie, Marshall, and many others, and reflects on the benefits they have conferred on medicine, surgery, and, above all, on the subjects of climate and the external causes of disease, that will refuse to the administrations of the medical departments of our fleets and armies their due importance, or allow themselves for a moment to view it as indifferent, whether men like Guthrie, after a career of the most brilliant success, shall be considered as "too young" for promotion, or a man of master-mind like Robert Jackson, shall be persecuted into retirement from the service, by corrupt or feeble administrators.

We consider it of the last importance that the Medical Departments of the Army and Navy should be well administered, not only on account of the interests of the public service, but for the sake of science all over the world. Is it of no consequence to the general welfare whether such men are to be elevated or depressed—whether they are to receive the just reward of their honourable exertions, or to be insulted and superseded by their inferiors? All we can say is, that such things, and even worse things, *have* occurred. We should be grieved indeed did we think it probable, or even possible, that they should ever occur again: but, without vainly diving into futurity, we will assure our naval and military brethren that, their best preservatives from a repetition of the ills that have gone by, will be found in their own general intelligence, aided by a knowledge of the particular subject now under consideration ; and last, though not least, by *union*.

The subject, however, is complex—" it comprehends a wide range of general and practical knowledge of military service, as well as a correct acquaintance with the history, causes, and consequences of the diseases to which troops are most liable, in the field or in quarters."

Such is the rough school* in which all must serve, who pretend, either as physicians or surgeons, to attend to the medical concerns of armies ; or further, who pretend to administer the affairs of a department involving these concerns. There is no other channel of information—no other school than this ; and it is now proved by sad and repeated experience that, where the " general and practical knowledge of military service," is wanting, there is no safety for the interests of the state, the welfare of the soldier, or the character of the medical establishments. We propose very briefly to shew, first, how this came about in the British Army,—but before doing so we would remark, once for all, that we adhere to the recital of bare facts—avoiding comment or censure.

The actors in the scenes are gone :—many of them were respectable persons ;—and, however careless and corrupt the system they administered, we believe the administrators to have been free from personal corruption.

From an early period till the year 1815, the affairs of the " Army Medical Department " in England were conducted by a Board consisting of three men. It never appears to have been considered necessary that these gentlemen should " at any period " have been " practically acquainted with the business " of their department :—yet by His Majesty's Warrant of 1798, when a reformation was instituted, the " Physician-General was to recommend the army physicians, and to give his opinion on all matters referred to him by the Commander-in-Chief, or Secretary at War ; that he was to inspect the medicines provided for the use of the army by the Apothecary-General, and to join with the Surgeon-General in checking his bills ; the Physician-General was also to certify his opinion in the

---

* " The surgeon of a regiment learns the duty of a soldier in addition to that of a doctor, and a military surgeon ought to know the one just as well as the other. I remember a village on the great plain of the Guadiana, near Merida, in which three regiments were quartered in the sickly season of Autumn, when fevers prevail. Three rows of hillocks marked the last resting-place of the dead on earth, and my attention was attracted by one row being much shorter than the other two. I found on inquiry, that the regiments were very much of the same strength, and quite under the same circumstances. The doctors were equally able ; two were men entering rather on the middle period of life, the third was a very young man, and perhaps the worst doctor of the three ; but the short row of tumuli belonged to him. I was very desirous of making this out, and after carefully visiting all the hospitals and quarters, I ascertained the reason. He was the better soldier, if not the best doctor. His hospitals were in better order, the material was more perfect, the labour bestowed on every part, except in physic, was greater, and five per cent at least of human life was the saving and the result. I never saw it otherwise."—*Guthrie on the Diseases of the Peninsula.*

cases of officers applying for leave of absence on account of ill-health, if the cases were not surgical; and he was to preside at the medical examination of candidates for regimental and staff commissions.

The duties of the Surgeon-General were to recommend staff and regimental surgeons and assistants; to select from the staff surgeons on full pay at home such as may be wanted for the general hospitals, camps, and districts in this kingdom; to make requisitions to the Inspector of regimental hospitals for apothecaries and hospital mates; and to appoint the inferior officers and attendants in the general hospitals. He was to correspond with the heads of hospitals abroad, and to be the channel of application for extending the leave of officers absent from such hospitals. He was to inspect the quality, and to regulate the prices of the surgical articles in the bills of the Apothecary-General, and, with the Inspector of regimental hospitals, to ascertain the claims for bounty or indemnification for loss of limbs, and cure of wounds. He was further to certify in surgical cases, as the Physician-General was directed in medical cases; and he was to assist at medical examinations of hospital mates.

The third principal officer of the medical department, stated in His Majesty's Warrant, Inspector of Regimental Hospitals, was directed to recommend apothecaries, purveyors and deputy purveyors, hospital mates, and the inferior officers, on the formation of any new establishment.

He was to inspect the regimental hospitals at home, to correspond with the regimental surgeons at home, and to be responsible for all matters relating to the supply of their medicines, and the management of their hospitals. He was to act with the Surgeon-General relative to the claims of wounded officers, to certify in surgical cases, and to assist at the examination of hospital mates."—*Fifth Report.*

How a Physician-General who "never served in any army"—a Surgeon and Inspector-General, who never served but in the Guards, confined to London—all three being private practitioners in London—conducted the difficult and important duties above described, we shall now relate. The Regimental Surgeons—the true physicians of an army—were proscribed "from the expectation of filling the physician's office, which was bestowed on hands of a high and privileged class, the physicians of the regular universities and associates of the College of Physicians of London," though almost as ignorant of the nature of army diseases as of the Welsh language, those of Oxford and Cambridge especially:—there existed an unparalleled, costly, and "unnecessary" proportion of officers, so that "two-thirds of the Medical Staff abroad during the last war were useless" (*Report*):—a wasteful, complicated, and injurious system of hospital management:—a defective plan of hospital record and return, affording no satisfactory or statistical result as to the nature of disease or the effect of treatment:—an unworthy use of patronage, granting commissions and responsible offices to persons "of inferior medical education," while the recommendation was in some cases with one member of the Board, and the responsibility for the conduct of the person recommended was with another member" (*Report*):—an assumption by one member of "very extensive patronage" belonging by His Majesty's Warrant to one or both the other members, "causing an unnecessary increase of many branches of the establishment, and a much greater expenditure than would otherwise have been incurred" (*Report*) :—the employment of persons " who, receiving the full pay and allowances of another office, did not execute those duties for which that pay and those allowances were given"—thus improperly " remunerating services of one kind by the pay and emoluments of a very different office" (*Report*) :—the permitting, contrary to orders, of large cash balances remaining in the hands of the Treasurer and Agent for Army Hospitals (*Report*) :—an expensive and unnecessary addition to the Staff of Hospitals at home and abroad—an addition " scarcely justifiable" (*Report*) :—the continuing " the same rank and pay" to purveyors when the rank " was no longer considered as a step of promotion in the medical line"—and when they had become " mere Storekeepers or Stewards" (*Report*) :—the particular " incongruous appointment, at the discretion of the Surgeon-General," of persons denominated " *Principal Medical Officers*"—persons who might be raised from any rank to this station, even from " an Hospital Mate," and so as to " supersede in control all his former superiors" (*Report*) :—the making " new appointments" at times when the Half-pay List could have furnished the requisite officers," and this contrary to " the rules laid down in His Majesty's Warrant" (*Report*) :—the continuance of the " very disadvantageous" system of the " General Hospitals" after their fatal tendency on military health—their profuse expenditure of money, and injurious influence on the medical character had been demonstrated :—" the fixing of complete establishments for General Hospitals at places where there were no patients," in a manner " unknown at the War Office" (*Report*) :—that " in consequence of the extension of the General Hospital system only" an enormous accumulation of surgical instruments, and loss of the public money took place, while, as to the medical stores of the army, which were often " very bad," " no public officer observed and took an account of the quantity and quality of the articles put into the packages," and it happened that it was only on their arrival at foreign stations that " deficiencies have been observed" (*Report*) :—" great inattention in the assortment of the medicines sent abroad," although it was obvious " that, for troops going to different climates, or to be engaged in services of different descriptions, a difference in the kind of stores to be provided may be necessary" (*Report*) :—the destruction of many public records that purported to complain of bad administration, a wasteful expenditure, bad medicines and bad instruments, the explanation being that " no trace of such Report can be found" (*Report*) :—the destruction of valuable stores of medicines and instruments from bad packing, from which " the public has sustained a considerable loss,"—also great inconvenience from " the contents of the packages not corresponding with the invoice put up with them" (*Report*) :—the permitting charges on account of Medical Stores, " 40, 41, and nearly 60 per cent higher" than the market prices of certain chemists and druggists of the day, although these latter " afforded something handsome in the way of profit ;" while a charge was allowed in full sets of surgical instruments of " 19 per cent," on portable sets of " 40 per cent. and on screw tourniquets, of 50 per cent," above Mr. Evans's charge : " and these prices have been allowed (as it would seem without inquiry) by those whose duty it was to check the charge," causing thus an expenditure of seven thousand pounds per annum on account of instruments alone, and that at a time " when it is known that the Regimental

Surgeons always found their own instruments" (*Report*) :—the establishment of " Depôts" at places where they were not wanted, and the filling them with expensive stores, most of which were left to decay; at Porchester Depôt alone there were " medicines sufficient for about 30,000 men, and Purveyor's stores for a much greater number;" while, at the York Hospital Depôt, there were 800 sets of capital instruments, with upwards of 1,300 full and small sets of pocket instruments, and at Porchester " 300" more sets of " capital instruments" (*Report*) :—the ordering of surgical instruments to the value of nearly eighteen thousand pounds in 1803, notwithstanding instruments to the value of eighteen thousand pounds were provided in 1801 and 1802, at the close of the last war;"—the result of the whole being that, " the annual expense of medicines in the navy had not amounted to above one-third of that of the army, although the naval hospitals were larger, and more numerous, than those belonging to the army, and the naval depôts were as widely extended" (*Report*) :—the absence of all check on the expenditure of " Hospital and Purveyor's Stores," though these last amounted to the enormous sum of " five hundred and eighty-five thousand pounds, or, on an average of thirteen years, to forty-five thousand pounds per annum"—thus exhibiting " the same inattention to economy in conducting the supply of these hospital stores, from time to time, as of the medicines and instruments" (*Report*) :—the causing to be transported from London many articles to the " different depôts, and from these subsequently to other depôts, at a great expense, that could have been procured on the spot where they would be wanted; often, probably, at a much less prime cost, and always, of course, without the other expenses which we have before enumerated" (*Report*) :—the total abandonment of the duty of control in regulating the quantity and prices of wine, spirits, and porter for the use of the army at home and abroad from 1793 to 1806, in a matter amounting to " forty-seven thousand, seven hundred and forty-nine pounds,"—the consumption in York Hospital alone being " a pipe of Port wine in ten days," the same rate of consumption being continued " for some time" (*Report*) :—it arose from the general system then in force, " 1st, that, owing to the variety of modes in which money was issued for the service of the department, its whole expense was never submitted in one view to the attention of the legislature: and, 2dly, that, owing to the variety of modes in which the different payments were controlled, no assistance was obtained for checking one species of expenditure by another, or of producing an uniformity and consistency in it" (*Report*).

We will not follow the " Report of the Commissioners of Military Enquiry" any further, or enter into the details of individual misconduct, but conclude with the dictum of the Duke of Wellington, pronounced after a long and intimate acquaintance, and which proved the death-warrant of the London Board :—" Of the Medical Board I entertain the very worst opinion. I have proof that every promotion is a matter of application and intrigue." The parties referred to are long since gone; and, after the exhibition already afforded, we do not apprehend that we shall again hear of a Board in England to conduct or superintend the medical affairs of the army.

It will be seen that the quotations are made principally from the "*Report*," and in some few instances from the works of Dr. Robert Jackson, whose

H 2

titles are inserted at the head of this article; for we consider both to be equally authentic. The carefulness and entire trueness of Dr. Jackson's observations are now well known, and were duly appreciated by the members of the Commission, who adopted them, "because they had frequent opportunities of ascertaining the correctness of the facts stated in them, and also because many of the improvements which have lately been made in the army medical system appeared to them to have been suggested by Dr. Jackson" (*Report*). All this is quite true; and we should like to see the character and services of this most upright and able military physician recorded in a manner worthy of them.

The biography of Dr. Jackson is a desideratum in medical literature. We should like to see also a manual for the guidance of the medical officers of the army, containing, in brief statistical form, an enumeration of the quantities and proportions of stores, establishment, &c., required for certain given numbers of troops in field and cantonment—likewise the probable number of sick per thousand of strength in certain climates. This latter information can now be obtained from the invaluable statistical Reports of Mr. Marshall and Major Tulloch.

Before concluding the subject of the former mode of managing the affairs of the Medical Department of the British Army, we would make a few observations on "The General Hospital System," which flourished systematically and especially under the Board, and was only overborne on the fall of the parent institution.

The destructive effects of general hospitals were early observed by our medical and military officers. "Among the chief causes of sickness and mortality in our army," says Pringle, "the reader will little expect that I should rank the hospitals themselves, though intended for its health and preservation, and that on account of the bad air and other inconveniences attending them." Dr. Robert Jackson is equally emphatic and more detailed in his account of their ill effects, and quotes the military of George the Second's time, who characterised this institution as "the destroyer of the army." The physical ills they produced by generating new diseases—tending to fatal relapses—counteracting recovery—causing delay in early treatment—and repressing the zeal of the Regimental Surgeons—were not surpassed by their moral ills, which went to destroy the best feelings of the man, and by consequence the best qualities of the soldier. In despite of former experience, however, general hospitals for the infantry were used on a large scale in Holland in 1794-95, and the mortality in them was excessive—ending in "dreadful destruction;" while the cavalry, which "traversed the same fields, and lived in the same air," but which carried its own sick, and treated them regimentally, "had little or no mortality in the whole course of its service." It was the same with some few corps of infantry that adopted the regimental plan of management. In the Parliamentary "Report," already so often referred to, the General Hospital System is described as "attended with most destructive consequences to the sick soldiers, and that it has produced expenditure and waste of every kind." Again:—we find, "the accumulated horrors of ill-arranged hospitals"—in other words, the terrible consequences of *hospital miasm*, deplored by the historian Alison. Treating of the Peninsular war, he says: "the military hospitals, charged sometimes with twenty thousand sick at a time, fostered contagion rather than cured disease,"

while the Government at home left its General no funds "to pay for hospital necessaries."—*Wellington's Despatches.* Let us hope, then, with Mr. Martin,* that we may "never again see the General Hospital System inflict our armies."

When, however, general hospitals must, of military necessity, be established, and for however short a time, their inmates should consist only of such sick and wounded men as cannot be removed; and crowding, above all things, ought to be avoided—the position, construction, and arrangement being, at the same time, of the most approved kind—the charge and control being conferred only on officers of the most approved and experienced character—in order that the various abuses, moral, medical, and financial, inherent in general hospitals, may be guarded against, so far as possible.

In conclusion, we will state that, previously to enlarging the accommodations of the Small-Pox Hospital in London, "erysipelas, typhus, malignant cynanches," and other formidable diseases were common: but "by spreading the same number of cases over double the extent of surface," these terrible consequences of crowding have been avoided. By those who have served so long as we have, it will not be said that we have dilated too much, or dwelt too long, on the medical management of armies. To them, "it is well known, that expeditions, apparently well concerted, have sometimes failed from sickness and mortality among the troops; and that campaigns, the plans of which seem to have been well laid, have had unsuccessful issue from the same cause. It is moreover true, that these effects have often arisen from defects in the original plan of medical arrangement, or from ignorance or inattention in the manner of execution."

But let not our readers suppose that they have yet done with us. There are still standing, or, we should more properly say, tottering, three Medical Boards in the East Indies, and we must just look back a little to the working *of their system*—first briefly adverting to the constitution of the Asiatic Boards. We are told by Mr. Martin, that they are strictly an institution of seniority:—that in Bengal at least, any man, no matter if he have been a manufacturer of indigo, silk, or salt-petre, or a holder of a civil or judicial office, may, by serving two years with a Sepoy battalion in cantonments in time of peace, render himself qualified, first for the office of "superintending surgeon," and then plump into a seat in "*The Board*:"—that "an individual of little worth, whether regarded as a medical officer or a gentleman, may now look upon it as certain that, provided he survives his contemporaries, he shall rise in due time, not only to the superior staff employs, but to the governing head of the service:"—that, notwithstanding "the principle of selection is paraded forth in the Regulations of the Service," there "have arisen, within the writer's recollection, not once, but frequently, the mere merchant, the confirmed gambler, and the exhaused tippler," so as to leave the rule of selection "a dead letter—a mere mockery:"—that "the members of the Board have, on an average, completed a period of forty years' service:—that they are necessarily superannuated, generally deficient in military expe-

* Johnson and Martin on Tropical Climates, page 572.

rience, and therefore cannot be expected to represent the feelings or the interests of the body of which the board is the nominal head:—that as members of a scientific corps, likewise, they represent an age gone by. "Under such a system," says Mr. Mangles of the Civil Service, "where every situation of pecuniary value is given upon a sort of tontine principles, as a bonus on longevity, and where, consequently, such situations are unattainable, out of turn, by the highest merit, and confer no distinction when attained; how is it possible that emulation, the mainspring of all useful and honorable exertion, should exist? How, again, is it possible that the three senior surgeons on the list should always be properly qualified to instruct, direct, and control the whole body of subordinate officers; how the next ten following names should always represent that number of efficient superintending surgeons? How would the affairs of the Revenue Department, to take an example out of many, be administered, if the Board were invariably composed of the three senior members of the service, and the ten next on the list had a claim of right to the commissionership?" This is unanswerable.

"Many of the evils of administration of affairs in general, in India," says Mr. Martin, "are traceable to a disposition to multiply checks so as in reality to cumber the executive machinery, thus casting the responsibility, not upon qualified individuals, but upon Boards. In the medical department a Board, instituted for the transaction of public business, consists, like the former Boards of the British Army, of three persons of equal power and authority; and as it rarely happens that three men think exactly alike in every thing, so, counterpoises existing, counteraction arises—movement is jostled, embarrassed—and effect is feeble or erroneous:—in short, a Board so constituted presents us with the very symbol of *vis inertiæ*. Judging by an extensive observation of more than twenty years, I should say that, of all the modes of perplexing simplicity of operation, of weakening energy, of nullifying unity of purpose, of clogging public business, and of evading substantial responsibility, a Board is the most direct mode; but especially a Board constituted on the seniority principle. * * * Were it as true in nature, as unhappily it is the reverse, that the best men live the longest, still the system of rise by the muster-roll would be the very worst. Repressive of all emulation in youth and manhood, and deferring promotion till the verge of life, it leaves for the discharge of public duty no energy, moral or physical, in the possessors of high office. Such would be the law of Nature in any climate; but it holds especially in tropical regions, wherein the course of life is notoriously precocious of old age."

We have now before us the composition of the London Board, and, at all events, the composition of the Medical Board of Bengal, if not of the other minor Presidencies. We have seen how the London Board administered the important and honourable trust placed in their hands by their Sovereign. In its composition it will rank, professionally, far indeed above any Indian Board; for, with exception to the able and distinguished Mr. Annesley, of Madras—the only trump ever turned up by the seniority system—and some few others of minor note, we are not acquainted with any Indian Medical officers of distinction who have lived to get out of a Board composed of men of from sixty to seventy years of age. On the contrary, all the men whose names have reached our shores have been, comparatively, young men; and so it ever will be—it is the course of nature. It must be so. Old men—old idle men especially—are behind us. It cannot be otherwise.

Sir Clifton Wintringham, Mr. John Hunter, Mr. Gunning, Mr. Keate, Sir Lucas Pepys, Mr. Rush, Mr. Knight and others, were celebrated men in their day; but we have seen how very bad, how very bad even on the part of the honest and great John Hunter, was the administration of the medical concerns of the army. Let us now turn to the medical administration in the East Indies. But before we do so, let us at once acquit the Indian Boards of all negligence or abuse in the audit of the great public accounts, and of any attempt at abuse of patronage, favouritism, or partiality in its distribution. In this verdict of entire acquittal on these great and important heads, we are sure our readers will unanimously concur with us, when we assure them—that, in India, our statesmen and financiers were wiser than their confreres at home; for, in the East, it never once entered into the mind of man to trust a Medical Board of seniority with such important and responsible duties as were here handed over to it. To the benefits of this plea then, in all its latitude, the Indian Boards are freely and fully entitled. Let us now look to the manner in which, in Bengal at least, they discharged the duties actually entrusted to them. We need not dive into musty old volumes of bad regulations, bad print, and worse paper, beginning with the year 1777, to learn what the duties of medical administration in an army are. Every body knows that these duties comprise the promotion and encouragement by every means in our power of every measure calculated to contribute to the soldier's efficiency, comfort and welfare, as well as to promote the honor, interest, and just administration of the service immediately under control. Tried by these tests, and they are the only true ones, we anticipate that the Indian Boards must be found wanting. First, then, as to the comfort and welfare of the soldier. We have before us a printed document, prepared, as we are informed, in Calcutta, in 1839, by two medical officers of the Bengal Army, and who must therefore have been cognitive of the facts and circumstances they state;—indeed they continually refer to official documents.

In this printed paper it is stated that, in March 1835, Mr. Martin, in his capacity of Presidency Surgeon, submitted to Government a detailed and extended plan for requiring from the medical officers of the three Presidencies of India and the dependencies to the Eastward, reports on the medical topography and statistics, of stations and cantonments with which each individual might, in the course of service, be best acquainted. This plan was referred by Government to the Medical Board, who reported on it in a manner to induce the Governor-General to believe they intended to "throw cold water on it." The plan, however, appeared to the Government to be good—so, in place of the hydropathic mode suggested by the Board, effect was given to the order for all India, *and that by a direct act of the Government, without further reference* to the heads of the Medical Department. "It will hardly be credited by the public or the profession in England that, when this plan came to be considered by the Medical Board of Bengal, it was attempted to be burked; the reasons urged by the Board (we quote their own words) being, that Mr. Martin's plan would seem to embrace a far wider range of investigation than is to be found recorded in the writings of Hamilton, Breton, Wade and others." The Board therefore, found itself "compelled to confess itself less sanguine in anticipating a favourable result from the zeal and industry of our brethren;"—a new mode this of eliciting the talents and energies of a whole service! But one word on the Board's *reasons* for clipping Mr. Martin's plan. " Hamilton wrote most ably, it is true, on political and statistical topography; Wade never wrote a line on any topographical subject whatever; and Breton but a detached report, in a periodical, of a particular district. For the reason, then that Mr. Martin contemplated a course 'far wider' than the persons named, must we have it smothered in a wet blanket!

" In May 1838, Mr. Martin, in continuation of his topographical plan, called the attention of the Medical Board to the importance of medical statistics, and particularly of that department of it termed hospital statistics, and the neglect of this latter in Bengal. Having explained at large the principles of what was required, he called the Board's attention to the condition of the General Hospital at the Presidency, (under the Board's immediate supervision) in which during the seventy years of its existence, tens of thousands of Europeans have been treated, yet not one table has been constructed from its records which throws the least ray of light on the numerous and important facts connected with the subjects of the influence of climate, of locality, of treatment, or indeed with the subject of hospital statistics in India; and he concluded by proposing the nomination of a committee of selected medical officers to inquire and report on this great question." But, continues the printed report—" If belief was staggered by the reception of his first proposition, it will scarcely be imagined that the one just stated was absolutely rejected! The Board, in its suicidal reply, after upholding the antiquated and useless forms of hospital returns now in use, and from which no statistical information can by any possibility be elicited, went on to maintain 'that the public interests will be most effectually promoted by every one attending carefully and zealously to the discharge of the duties entrusted to him.' This rebuke was bestowed on the proposer of the plan, at the very time when the functionary who concocted it was writing daily in the newspapers on the *Judicial System* of India, or dedicating immortal poetry to 'the people of Scotland.' Such was the juncture chosen to tell Mr. Martin that the Medical Staff of the army might drug their men ad libitum (croton oil for congestions, and salts for Sepoys); but as for medical topography and statistics, they were in the Board's keeping. "Will it again be credited that the Medical Board, who threw cold water upon Mr. Martin's plans, have since taken up the subject themselves, after a fashion of their own, thus exhibiting less of consistency, singleness of purpose, and public spirit in the matter, than of anxiety to appropriate to themselves the amount of credit that may accrue from eliciting medico-topographical and medico-statistical results from different quarters; and reducing them to their proper place as elements of science."

But the Board *did* bring out their own fashion of a return; and here is the explanation it called forth:—" Note presented to the Governors of the Native Hospital of Calcutta, by Mr. Martin, Surgeon to the Institution, dated April 15th, 1839."

" 1st. It may be well to observe, before entering on particulars, that, for the purpose of obtaining true statistical information in systematic form relating to military and other hospitals, it is everywhere necessary that there shall be different classes of tables—some elementary, others more and more complicated, until the end of a term is reached, such as a year, when the whole is compressed into a final and comprehensive table, such as the annual one in use in Her Majesty's Military hospitals.

"2nd. The advantage of such organised plan is obvious; and, without it, all the labour of constructing tables can only prove an insupportable labour to the compiler, for the reason that he is not cheered on by an interest in the subject, or by any prospect of ultimate utility.

"3rd. The accompanying formula transmitted by the Medical Board, contains, I regret to say, nothing from which any useful information whatever can be framed, and this opinion I know to be entertained by officers of the first rank and experience. In it are comprehended observations elementary and ultimate, all so blended together that, by no contrivance that I am acquainted with, can the true statistics of disease be obtained. Matters too, that ought to be recorded in the daily, weekly, monthly, quarterly, and annual returns, are here mixed up in one page, so as to confound all attempt at exploration. * * * The opinions I here reluctantly, but respectfully, venture to express, are borne out by the admirable exposition of the system of hospital records in use, and which are altogether worthy our imitation, in Her Majesty's Service, as presented to the Municipal Committee, by the Inspector-General Macleod.

"4th. The nomenclature, also, of the Medical Board's formula will be found defective, as compared to that in use in Her Majesty's hospitals, a copy of which I subjoin :—again, invalidings, which never occur but once a year, are here introduced into a Monthly Return for European Hospitals, along with another calculation which is only used annually. * * * Many other objections might be adduced to the entire plan and construction of this formula, such as the first and last items in the table, and the errors found by the President of the Municipal Committee to belong to them: the item 'other diseases,' too, is highly objectionable in any report which aims at statistical accuracy. But I shall not further occupy the time of the Meeting, by noticing these matters; and I can assure the Governors, that nothing but a sense of duty to the Institution has caused me to submit the above remarks."

Here, then, we have exhibited the conduct of the Board in a manner not to be questioned. If it be alone through the cultivation of medical topography and statistics that we can arrive at an accurate knowledge of the influence of climate and of locality on military health—of the advantages of different modes of medical management—of accommodation,—of diet, clothing, occupation and mode of conducting duty, &c. &c. on the part of the soldier ;—if, in short, these be the sole channels of inquiry into, with a view to ameliorate the moral or physical condition of the soldier, what are we to think of the Bengal Board's conduct? Was it promotive or obstructive of the measures proposed? Was it, to use the Board's own words, discharging the duties intrusted to them in the most effective manner for the public interests? We will allow our readers to answer this question.

We have no pleasure in passing censure, however merited; and we have purposely avoided comment throughout this article—choosing rather to let the public decide for itself.

It was in vain that Mr. Martin urged upon the Board the importance of the subject, in tropical climates especially, and the necessity there existed of entering upon it in a systematic form—urging, in support, the sentiments of the ablest men in England. The Board would not move. It was in vain that Dr. Bostock, and others of the first class of eminence in Europe, declared "the numerical method" to be "one of the greatest improvements of modern science." The Board was deaf. Strange as this conduct will sound in European ears, Mr. Martin, in another place, has given us what we believe to be the real clue to its interpretation:—"We are, in India," he says, "continually kept in mind of that law of our nature, by which old men are disinclined from undertaking any thing, however excellent, of which they cannot be expected to see the end. Through the operation of climate, also, we have too often to lament the premature display of the contracting influences of age on the moral and physical constitution of man, to the prejudice, no less of public welfare than of private happiness." But, though the Board rejected Mr. Martin's plan for carrying out a measure of great prospective advantage to the State, because they could not be expected to see the end of it, they entered warmly into a measure of questionable utility it is true, but presently involving their own personal comfort.

*From the Calcutta Newspapers of May*, 1841.

"INSUBORDINATION AT THE MEDICAL BOARD."

"There is a somewhat novel state of things, regarding the Members of the Medical Board, at present under the consideration of the higher authorities, and which, immediately relating to the question of military uniform, involves the higher one of military authority. We shall relate one of several accounts, (not substantially varying,) which we believe to be the most correct.

"Not very long since, Mr. Sawers, the senior member, considered of a sudden that as there was a uniform for the Medical Staff, that uniform should be worn at all meetings of the Board, and he mentioned this desire to the other, or junior, members, Doctors Campbell and Smith, and said at the end of a fortnight (allowing that time for the uniforms to be prepared) they should appear accordingly. They, considering this as a proposition, rather than as an order, voted against it, and intimated to Mr. Sawers that his motion was negatived by the majority.

"He made no remark whatever upon this result, and such meetings as next ensued, were attended in the old way—plain cloth coat, or white jacket, according to the 'warm feelings' of the respective members—until the first meeting occurred after the expiration of the fortnight's law,—when, on Dr. Campbell's entering the office in a white jacket, Mr. Sawers, who was himself in undress uniform, ordered him to go home, and consider himself in arrest for disobedience of orders.

"Home he went accordingly, and there he has remained in arrest ever since, and charges have been sent in against him by Mr. Sawers, grounded on his recusancy. These charges are before Government and the Commander-in-Chief, and we understand it is not found an easy matter to decide how they should be dealt with."

Let us imagine three gentlemen, whose aggregate age may be taken at 200 years—that is, the senior of them being taken at 70, and the two juniors at 65, ordered, in defiance of age and the functions of the human skin, and under pain of military arrest, to carry on their deliberations clothed, under a temperature of 93°, and in the rainy season in Bengal, in uniforms of scarlet woollen cloth, bound in ominous black velvet—the whole surmounted by huge cocked hats and black feathers. Sitting in this fashion of military undertakers, let us imagine their sufferings, which caused a delicate West India lady, decked out in gauze muslins, to compare her condition to "the feel, as if she had been bathing in a boiler of syrup." Those alone who survived the agonies of the "Black Hole," could duly estimate the perils attending such mode of deliberation.

But we must now leave the Board enveloped in its own vapour, and turn to the manner in which "the honor, interest, and just administration of the service immediately under control," have been looked to. As to honors and honorary distinctions, they are soon disposed of. There are no such things in the Medical Departments of India; and while nearly sixty officers of the Bengal army alone, are distinguished by honorary insignia, not one of the medical officers who served in the same ranks, and incurred more than their share of risk from various contingencies, has been so marked out. It is now statistically proved that the average mortality of the medical officers all over India is greater than that of the infantry branch even; and the Burmese and Affghan wars, not to speak of the earlier campaigns of India, attest the increased casualties consequent on active military service. As to the interests of the department—a very cursory inspection of Mr. Martin's "Notes," will prove that neither these nor the just administration have been in the least degree regarded by the constituted heads of the service.

"During the last sixty years, the formation, discipline, and economy of the Indian army have been variously and advantageously modelled upon those improvements, the suggestions of experience which have been adopted in the Royal Service: whereas the Indian Medical Department, an integral part of the army, has, by a strange oversight, been left just where that of Her Majesty's Service was in 1780:—in other words, it has remained stationary during these sixty years; and, up to this hour, it is deemed to have no claim to any other mark of distinction than is included in the mere routine promotion by seniority:—in fact, the stagnation of this anomalous principle weighs upon it with peculiar and deadening effect, especially in Bengal, where promotion is so far more slow than in the other Presidencies. The great body of the service—debarred by legitimate expectation, and hope of rise or distinction, beyond such as belongs to a name borne so many years on the muster-roll—rather than to a character for knowledge, activity, and discernment—droops and stagnates, so as, after a time, to lose that salutary zeal which prompts to those exertions that, in all other services, secure a just and honorable reward. Disadvantages press on every grade of the service:—they comprise exclusion from the honors and privileges attached to military advancement—a low military rank—a too low rate of retiring pension, as well as the want of a greater number of grades; in short, the entire service demands a remodelling, to keep pace with the general improvement of the Indian Army, and to assimilate it to the Medical Department of Her Majesty's Army."

Now to the proof—

"There are but four grades in the Company's Service, viz, the assistant-surgeon ranking with a lieutenant—the surgeon with a captain—the superintending surgeon with a lieutenant-colonel—and the member of the Medical Board with a colonel. We have no grade equivalent to that of major—a subject of complaint for many years, on account of its manifest injustice in a variety of ways. * * * In the present condition of the Indian Medical Department—deprived of grades and honors—an officer, when he has attained the rank of surgeon, may consider that, for the next twenty years, he has nothing further to hope, and that promotion will reach him only with superannuation."

Again—

"On attaining the long-envied rank of superintending-surgeon, the incumbent must serve two years before he can lay claim to the pension of his rank; whereas the military officer has only to be Gazetted to possess his right of pension for life from the date of such Gazetting. This is considered a peculiar hardship in the medical service, and with justice, it is believed, seeing that, at present, the ten senior surgeons on the Bengal list are of thirty years' service, or upwards, and who, according to existing rules, are entitled to no higher rank or pension than that of captain, viz. £191. per annum.

"In the Royal Army the rate of pay progresses according to the date of standing in the service, and, by its pension rules, twenty years passed in the East Indies are equivalent to thirty in the other British dominions."

Here then we have exhibited a state of things in very humiliating contrast with that which has long held in Her Majesty's Service. Now for a contrast of the manner in which the interests of the Medical Services of India have been attended to, as compared to those of the Indian Army.

"Let the following facts speak for themselves:—a captain retires after completing twenty-four years actual service in India, on major's pension, or £292; a captain of twenty-eight years service on lieutenant-colonel's pension, or £365; —a captain of thirty-two years service, on colonel's pension, or £456:—while a surgeon of twenty-four, twenty-eight, and thirty-two years has yet but a pension of £191 for a service solely in a tropical climate; and we have now, in Bengal, surgeons of thirty-three years' standing unpromoted. Again:—one-third of the colonels of the army have regiments, affording a competency for life; and of 191 lieutenant-colonels and majors, one in two is in the receipt of extra-regimental allowances for command of corps. Of the field officers besides, fifty-six, or one in five, have staff situations with handsome salaries; and of the captains, 159, or one in three, hold similar advantages; while 389 lieutenants, or one in two and-a-half, hold staff offices. In addition to all these advantages, the officers of the army have the privilege of purchasing out seniors to an unlimited extent.

"Now, of 106 surgeons in Bengal, only ten hold Staff appointments with allowances above regimental surgeons; and of 244 Assistant Surgeons, 14, or 1 in 18 only, have the same advantage, while in the army of the same presidency, one officer in six holds field rank—and only one surgeon in twenty-six stands in corresponding position. The comparative disadvantages under which we labour," concludes Mr. Martin, "are as numerous as they are impolitic in themselves and humiliating to our service."

This is quite true: and now let us ask, how it was that, during these sixty years of neglect and abuse of every kind, the constituted heads of the Indian Medical Department remained silent and inactive? How has it come about, also, to quote the Lancet, that "not one of the many wealthy old men whom the Indian Medical Service has sent home, has had the good feeling, or knowledge enough of the subject, to bring the condition of his brethen, whom he left behind, to public notice?" We leave the Boards to answer these questions. We know that, to the several memorials recently sent home, we had, as in duty bound, a seniority catalogue of names duly registered according to the muster-roll, and beginning with the first three names in the list, these by the next ten, and so on: but what became of these said memorials? Who read them? What respect or consideration did they meet with on the score of the seniority list of signatures? Was any thing ever done that they petitioned for? We again leave the Board to answer. When men who had passed their lives in idleness, neglect, or in absorbing non-professional pursuits, rose to the administrative heads of the medical department of the army, when and where did we hear of remonstrances from the Boards?

When the mere merchant, the confirmed gambler, and the exhausted tippler got into a Board, who ever proposed to turn him out? When the whole body of the service laboured under the most shameful neglect, disability and injustice, what Board in its corporate capacity ever stepped forward to its aid? To all these questions we should like to have an answer. When measures calculated for the good of the state, the soldier and the service at large were proposed in the most respectful manner, what was the conduct of the Bengal Board? The public can now answer to these matters. When, in an official Report, published by order of Parliament, it was stated that the mode of "Return" in use in the Indian army was defective in every essential information and object tending to promote the health and efficiency of the soldier, did the Boards take measures to correct the important error complained of; and lastly:—When it was proposed, because of this neglect, to supersede and injure most materially the whole body of medical officers of India, did the Boards take any and what steps to support the honor and assert the rights of those under their immediate control?—Again we leave the Boards to answer.

Doubtless many of the evils here enumerated are inherent in that worst of institutions—a seniority promotion—and come back upon us by a sort of "natural retribution" from its long continuance: but, though we may therefore be disposed to pass a less severe sentence on the actors, we thus only acquire the more just claim to condemn and put down the ruinous and iniquitous system.

It is something glorious, says the Lancet, "to strike, as Mr. Martin has done, a death-blow at this vile 'system.' We will venture to say that it is something very useful; and if Mr. Martin should live twenty years longer (by which time we believe he would, by the rule of the muster-roll, be entitled to a seat in the Bengal Board) we are well convinced he will have no objection to stand by the test of comparison in utility with any stickler (if any should then be found alive) for the rule of absolute rise by seniority. All public men and public measures will be tried by the result—as Napoleon has well expressed it—the rigorous law of history. To the result then we may with safety appeal; and while all other matters are taking the course which justice and the spirit of the age prescribe, let our Indian brethren enjoy the substantive goods procured them, in the shape of pensions, by Mr. Martin's exertions: for now, unpromoted surgeons will receive £300, £350, £500 and £700—no one of whom formerly had but £191 per annum. This is 'something very useful,' we trust; and the rest will follow in due time. Meanwhile, let the medical services of India consider well, after careful examination, what the medical department of an army ought to be—what it has been—and what it now is in the Royal Service, under the honest, able and judicious administration of one responsible head—'openly and solely responsible for his own acts.' Let them consider what this latter mode of administering the medical affairs of the British Army has done for the welfare of the soldier, the honor of the service, and the advancement of science. When our brethren have well considered these matters, we have no doubt how they will act. No where does there exist a more able body of officers, taken as a whole, than in the Indian medical departments—and no where have medical men more varied or more extensive opportunities. That more has not hitherto been made of those opportunities is solely the fault of administration—of a false position. 'The medical service of India,' says Mr. Martin, 'claims the consideration of authority, on the score of its skill, labour, and activity, which have never been confined to mere recognition of professional duty, but extended to the developing the resources of the empire at large, and to the moral improvement of its vast population. In these respects no class of the Company's servants has a higher claim to the consideration of the ruling power.' Again, he says truly—'the talent is ample, but the prize for exertion has never been held forth.' "

With one more recommendation from their brother-officer, we conclude:—" *let them observe and record in early life.*" This rule, though opposed to the principle of the seniority system, they will find to their best advantage. It comes from one who has served " long enough to know, why the soldiers of the Tenth Legion were attached to Cæsar;" and what, perhaps, is more to the present purpose, how, by a just regard to the interests of the soldiers of the nineteenth century, we may secure at once attachment from them—advantage to our country, and honor to ourselves.

---

*Postscript.*—We beg leave to say a parting word or two on military ranks and honors.

Amongst our brethren of the army and navy, we know that, on the question of their right to both ranks and honors, there exists but one opinion; and this opinion rests on grounds of the most unquestioned justice. Robert Jackson says that, " as the medical staff shares in the fatigues and dangers of war, in just reason it is entitled to a share of advantages;" * and so thought and acted Napoleon—" Napoleon, the greatest man of whom history makes mention—Napoleon, the most wonderful commander, the most sagacious politician, the most profound statesman," was glad to place his best military surgeons in the same class of the Legion of Honor with his best generals. How would he have acted towards a man like Guthrie (to take an example out of many?) Would he have allowed such a man to remain undecorated? Would he have considered his claims to military rank and honor inferior to those of a man like General Dupont? Would he have considered him as less worthy of distinction than a captain of a gun-brig, or a lieutenant-colonel? Would he have permitted some of the most ordinary common-minded men that walk the earth, whether considered as soldiers or citizens, to be oppressed and overwhelmed with decorations, while a man like Guthrie stood forth undecorated? He would have done none of these things. It may be

---

* " It is quite impossible for a regimental surgeon to be out of fire, if he does his duty: and a medical staff officer can scarcely be out of the way of cannon-shot. The doctors are, therefore, very unjustly treated in being classed with clergymen, and commissaries. They do not actually fight, it is true, but I will venture to say, that the surgeons of the Fusilier Brigade in Spain, have been under more fire, take it all in all, than a large proportion of the general officers of the army."—*Guthrie's Lectures.*

well that on such a subject, however, we have the French Emperor's own reasons:—" In the council held to frame the Code Napoleon, it was proposed by Count Mathieu Dumas, that the order of the Legion of Honor should be confined to military men."

" Such ideas," said Napoleon, " might be well adapted to the feudal ages, when the chevaliers combated each other man to man, and the bulk of the nation was in a state of slavery; but when the military system changed, masses of infantry and phalanxes, constructed after the Macedonian model, were introduced, and after that, it was not individual prowess, but science and skill, which determined the fate of nations.

What is it now which constitutes a great general? It is not the mere strength of a man six feet high, but the *coup-d'œil,* the habit of foresight, the power of thought and calculation; in a word, civil qualities, not such as we find in a lawyer, but such as are founded on a knowledge of human nature, and are suited to the government of armies. The general, who can now achieve great things, is he who is possessed of shining civil qualities; it is the perception of the strength of his talents which makes the soldiers obey him. * * * * We must not reason from ages of barbarity to these times. France consists of 30,000,000 of men, united by intelligence, property, and commerce. Three or four hundred thousand soldiers are nothing in such a mass; not only does the general preserve his ascendancy over his soldiers, by civil qualities, but, when his command ceases, he becomes merely a private individual. The soldiers themselves are but the children of citizens. The tendency of military men is to carry every thing by force; the enlightened civilian, on the other hand, elevates his views to the perception of the general good. The first would rule only by despotic authority, the last, subject every thing to the test of discussion, truth and reason. I have no hesitation, therefore, in saying that, if a preference was to be awarded to one or the other, it belongs to the civilian. If you divide society into soldiers and citizens, you establish two orders in what should be one nation. If you confine honors to military men, you do what is still worse, for you sink the people into nothing."

" Moved by these profound observations," says Alison,* " the council agreed that the proposed honors should be extended indiscriminately, to civil and military distinctions." " While, up to this day," continues Mr. Martin, " the government of England considers such as the Earl of Chatham, Generals Whitelock and Prevost, more deserving of military honors, than men like Pringle, Blane, Robert Jackson, Macgrigor, Guthrie, Burnett, James Johnson, William Fergusson, Hennen, and a host of distinguished officers, who have encountered every kind of danger, in all climates and situations, and who have conferred incalculable benefits on the fleets and armies of England, as well as on mankind at large."

Theoretically, England would be ashamed to admit—practically, in free and commercial England, no one would dare to avow—" the influence of the mustache on the reason, and the necessity of the sabre in Govern-

---

* History of Europe.

ment:"—yet does she, in respect to many of her most meritorious servants, act in a manner less just, less prudent, and less liberal than did Imperial France—nay, than does at this moment any European government with which we are acquainted. It may be very true that, morally, men like Guthrie, stand forth well distinguished in the simple attire of a citizen, along side of blazoned heroes like Sackville, Chatham, Murray, Prevost, and others, whom we could count by the score. But this kind of distinction is not the one under consideration :—it is one too lofty for Governments ;—it is not attained by the wearing a badge. What we contend for is justice; and for the Government of England to be just and liberal in this matter, is most easy. It would cost nothing in money :—it would set aside unjust and humiliating distinctions. All this it would do, and nothing else.

